# Cell density-dependent ectopic expression in bloodstream form *Trypanosoma brucei*

**DOI:** 10.1016/j.exppara.2013.03.017

**Published:** 2013-06

**Authors:** Moazzam Ali, Mark C. Field

**Affiliations:** Department of Pathology, University of Cambridge, Tennis Court Road, Cambridge CB2 1QP, UK

**Keywords:** *Trypanosoma brucei*, Ectopic expression, Ribosomal RNA promoter, Cell density

## Abstract

Ectopic expression of either wild type or mutant proteins is a standard method in cell biology, and a vital part of the tool kit of molecular parasitology. During study of protein expression levels mediating intracellular trafficking, we became aware of highly variable expression between experiments. When investigated systematically it became apparent that ectopic expression of proteins from a ribosomal promoter diminished at high cell culture density in bloodstream form *Trypanosoma brucei*. This phenomenon was not restricted to expression of a specific protein or cell line or the vector backbone. While procyclic form cells did not exhibit detectable density-related expression changes, bloodstream form cells manifest significant reduction in expression at high density, confirmed by qRT PCR, Western blotting and fluorescence microscopy. Culturing in conditioned media unveiled a similar reduction in expression at lower cell densities. Taken together we concluded that this effect is likely related to the influence of a diffusible factor present in conditioned media and has implications for accurate quantification of ectopic expression using transgenic expression systems.

## Highlights

• Ectopic expression is a standard tool for molecular cell biology in trypanosomes.
• Ectopic expression of a range of proteins derived from rRNA promoters depends on cell density.
• The effect on expression is potentially mediated by a diffusible factor.
• Density-dependent expression was not observed in insect stage parasites.

## Graphical abstract

**Figure:**
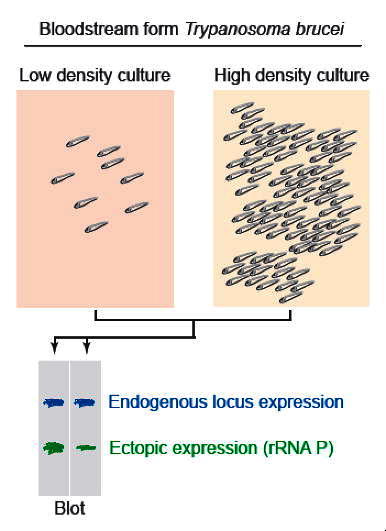

## 1 Introduction

Ectopic expression is one of the most widely exploited tools for the functional analysis of gene products in cells, for example, by over-expression of a protein to elicit a phenotype or expression of mutant forms of proteins to specifically block processes and map sequence to function. Further, this is a strategy that is convenient where antibodies are unavailable and/or genomic tagging is not possible due to low endogenous expression or other issues. Ectopic expression is generally assumed to be constitutive under most experimental conditions, but is rarely analysed in detail. Frequently, such expression is driven by constitutive promoters inserted into the genome in regions distinct from their original location, and the assumption is that these elements act as essentially isolated units, being designed to encompass all splicing sites and mRNA processing signals required. If variable expression is required inducible systems are employed.

In trypanosomes, while the range of reagents available is substantially fewer than mammalian cells, an impressive range of plasmids have been produced by many groups to facilitate both constitutive and inducible ectopic expression (Quijada et al., 2002; Wirtz et al., 1998; LaCount et al., 2002; Kelly et al., 2007). This strategy is part of the standard toolbox for the study of gene and protein function in trypanosomes, and has been exploited extensively. As part of recent work we have become specifically interested in analysis of the effects of over-expression or RNAi on proteins in trans, i.e. analysing the effect of alteration of copy number of one gene product on copy number of a second (e.g. Lumb et al., 2011) to infer functional interaction. We have also been addressing effects on turnover and protein stability resulting from manipulation of a second protein (e.g. Leung et al., 2011). In many cases the analysed protein is ectopically expressed to overcome issues with poor detection from suboptimal antibodies or low endogenous copy number, as well as to avoid, in many cases, generating an antibody reagent for a limited set of experiments. Again, this is also a standard approach in studies in model organisms and parasites.

During investigations of the functions of small GTPases in the endosomal system, and Rab21 in particular (MA and MCF, in preparation), we observed highly variable expression of various reporter proteins. When analysing localisation such issues are either unimportant or non-apparent. For example, in mammalian cells transient transfection is frequently used and results in heterogenous expression levels across a population of cells. However, in trypanosomes, transfectants are frequently cloned, so that clonal variability becomes more prominent. As this phenomenon was of potential significance to our ongoing studies we chose to investigate this phenomenon in using a systematic approach. Specifically, we asked if the effect depended on the specific protein being expressed, if this were due to a specific vector or if the phenomenon depended on the life stage being considered. Finally we sought to determine the conditions that led to the effect.

We found that ectopic expression in *Trypanosoma brucei* bloodstream stages, driven by an rRNA promoter, was reduced significantly with increasing culture density. This was not because of a generalised transcription/translation arrest, as endogenous short half-life control protein expression levels remained unchanged. The rRNA promoter is widely used for constitutive ectopic expression and this observation is therefore of general interest to the field of *T. brucei* cell biology and genetics.

## 2 Materials and methods

### 2.1 Culturing of *T. brucei* cell lines

*T. brucei* bloodstream form cells (Lister 427) were cultured in HMI-9 supplemented with 10% fetal bovine serum, 100 U/ml penicillin, 100 U/ml streptomycin and 2 mM l-glutamine) at 37 °C with 5% CO_2_ in flasks with vented caps (Hirumi and Hirumi, 1989). Procyclic form cells were cultured in SDM79 supplemented with 10% fetal bovine serum, penicillin, streptomycin, l-glutamine and haemin (Brun and Schonenberger, 1979). Ectopic expression was maintained using antibiotic selection. The CELLSPIN system from Integra Biosciences, Switzerland was used to agitate BSF cultures. 1 l spinner flasks were used to spin the cell culture at 50 rpm in a standard cell culture incubator. Cell density was counted using haemocytometer or Z2 Coulter Counter (Beckman Coulter), averaging three measurements.

### 2.2 Nucleic acid extraction

For genomic DNA isolation from BSF cell, 1.5 × 10^7^ cells were harvested by centrifugation, followed by washing with PBS. The cell pellet was resuspended in 300 l of TELT buffer (50 mM TrisCl pH 8.0, 62.5 mM EDTA, 2.5 M LiCl and 4% v/v Triton X-100) and mixed with 300 l of phenol:chloroform:isoamyl alcohol mix (25:24:1, v/v/v Sigma). The aqueous phase, separated by centrifugation was mixed with 0.7 volumes of isopropanol to precipitate DNA. For RNA extraction 2 × 10^7^ *T. brucei* (BSF or PCF) cells were harvested by centrifugation at 800*g* for 10 min. The cell pellet was washed once with PBS and snap frozen in dry ice. Total RNA was purified using an RNeasy Mini Kit (Qiagen) according to the manufacturer’s instructions. RNA concentration and purity was determined using a NanoDrop spectrophotometer (Thermo Fischer Scientific).

### 2.3 Quantitative reverse transcription PCR

First strand cDNA was synthesized using SuperScript™ III Reverse Transcriptase (Invitrogen). Quantitative real time PCR was performed using iQ™ SYBR Green Supermix set on a MiniOpticon Real-Time PCR System, both from Bio-Rad. Primers were; qRT YFP F CGACCACTACCAGCAGAACA, qRT YFP R GAACTCCAGCAGGACCATGT, qRT mCherry F CCCCGTAATGCAGAAGAAGA, qRT mCherry R CTTGGCCATGTAGGTGGTCT, qRT Rab 21 F CGTTCCATTGCTTAACAGTGAC, qRT Rab21 R CACATCGTAGACGAGGATTGC. All other primers were as described previously; TbVps23 (Leung et al., 2008), TERT and PRF2 (Brenndorfer and Boshart, 2010). The normalised expression (ΔΔCt) of mRNA was determined using Bio-Rad CFX manager software.

### 2.4 Generation of transgenic *T. brucei*

For ectopic expression pHD1034 or pXS5 were used to clone fluorescent protein genes or N-terminal epitope tagged proteins. pHD1034 was used for overexpression in both life forms while pXS5 is specific for BSF. For ectopic expression TbRab21 was amplified from genomic DNA using primers RAB 21f TAAAGCTTCTCGGTCGTCGGTGCCTAGT, RAB 21r CGGATCCTCAGGAACAACAGCGGTTC and cloned into pHD1034 containing an N terminal HA-tag. Expression constructs for YFP, mCherry and HA-TbVps23 were described earlier (Leung et al., 2008; Lumb et al., 2011). *T. brucei* bloodstream form cells were transfected with 10 μg NotI linearised plasmids. An Amaxa Nucleofector™ device was used for transfection together with the Amaxa Human T cell nucleofector kit. Cells were selected for 5–6 days using puromycin 0.2 g/ml, and/or phleomycin 1 g/ml as appropriate (Field et al., 2008). PCF cells were transfected as described (Hall et al., 2004).

### 2.5 Protein electrophoresis and western blotting

For lysate preparation, cells were harvested by centrifugation at 800*g* for 10 min at 4 °C, washed in PBS and resuspended in SDS sample buffer before heating at 95 °C for 10 min. Samples were electrophoresed on 10% SDS–PAGE minigels at 5 × 10^6^ cell equivalents per lane and then transferred to PVDF membranes (Millipore) by wet blot in transfer buffer (192 mM glycine, 25 mM Tris and 20% (v/v) methanol). Ponceau S staining was performed to monitor loading, by shaking membranes in Ponceau S solution (0.1% Ponceau S in 5% (v/v) acetic acid, Sigma) for 5 min and washing off excess stain with Milli-Q water.

Membranes were blocked and processed following standard procedures. Primary antibodies were used at the following dilutions; Rabbit anti-GFP (Abcam) 1:20,000, mouse anti-HA (Santa Cruz Biotechnology, Inc) 1:2,000, rabbit anti-ISG75 (from M Carrington, University of Cambridge, UK) 1:10,000 and rabbit anti TbRab11 (Jeffries et al., 2001) 1:2,000. Secondary antibodies; peroxidase goat anti-rabbit conjugate or peroxidase rabbit anti-mouse conjugate (Sigma) were used at 1:20,000. Bound antibodies were detected by reaction with luminol and visualized by exposure to Kodak BioMax MR X-ray film or using a G:BOX CCD chemiluminescence imaging system from Syngene. For quantitation either the G:BOX or film exposed within the non-saturated range was used.

### 2.6 Metabolic labelling

For ^35^S metabolic-labelling, BSF cells expressing YFP were grown to different culture densities and harvested by centrifugation (800*g*, 10 min). Cells were washed in Met/Cys free RPMI-1640 medium supplemented with dialysed FCS and incubated for one hour at 37 °C. Cells were brought to a density of 1 × 10^7^/ml and labelled with 30 Ci/ml [^35^S] EasyTag™ express protein labelling mix (Perkin Elmer) for 10 min at 37 °C. After labelling cells were washed briefly in PBS, lysate prepared and proteins separated by SDS–PAGE. Gels were fixed (10% acetic acid and 30% methanol (v/v)) and stained with Comassie blue before impregnating with scintillator by soaking in En^3^Hance (Perkin Elmer) following the manufacturer’s instructions. To detect radiolabelled proteins, gels were dried and exposed to Kodak BioMax MR film for several hours at −80 °C.

### 2.7 Fixation of trypanosomes for microscopy

BSF and PCF cells were harvested by centrifugation at 800*g* for 10 min at 4 °C. Pelleted cells were gently washed with ice cold Voorheis’s modified PBS (vPBS; 136 mM NaCl, 3 mM KCl, 16 mM Na_2_HPO_4_, 3 mM KH_2_PO_4_, 40 mM sucrose, 10 mM glucose, pH 7.6) (Nolan et al., 2000) and fixed for 10 min for BSF or 60 min for PCF on ice in 3% formaldehyde made from paraformaldehyde. Cells were adhered to poly-lysine coated slides (Polylysine™ VWR International) and coverslips mounted using Vectashield (Vector Laboratories, Inc.) mounting medium supplemented with 4′,6-diamidino-2-phenylindole (DAPI). Coverslips were sealed with nail varnish (Max Factor) before observing under a Nikon ECLIPSE E600 epifluorescence microscope. Images were captured using a Hamamatsu ORCA charge-coupled device camera. Digital images were analysed and false coloured using MetaMorph 6.0 software (Universal Imaging Corporation) and figures assembled from raw images using Adobe Photoshop CS6 (Adobe Systems).

## 3 Results

### 3.1 Decreased expression levels of ectopic constructs in high density cultures of trypanosomes is a general phenomenon

In initial experiments designed to address the influence of trypanosome Rab21, a small GTPase, on the copy number of a number of proteins in trans, we analysed whole cell lysates following RNAi against Rab21 (MA and MCF, in preparation). Data were highly variable, despite efforts to minimise experimental and nonsystematic errors, as well as the use of internal controls. It became apparent that this variability was not due to experimental variability, but rather was of biological origin. Hence, to systematically investigate these variable expression levels in cells, we devised a strategy to determine if the effects were vector or protein dependent. To this end, several fluorescent or epitope-tagged proteins were expressed in *T. brucei* and their expression levels quantified using a variety of methods, to eliminate systematic errors with the quantitation technique.

**Fig. 1:**
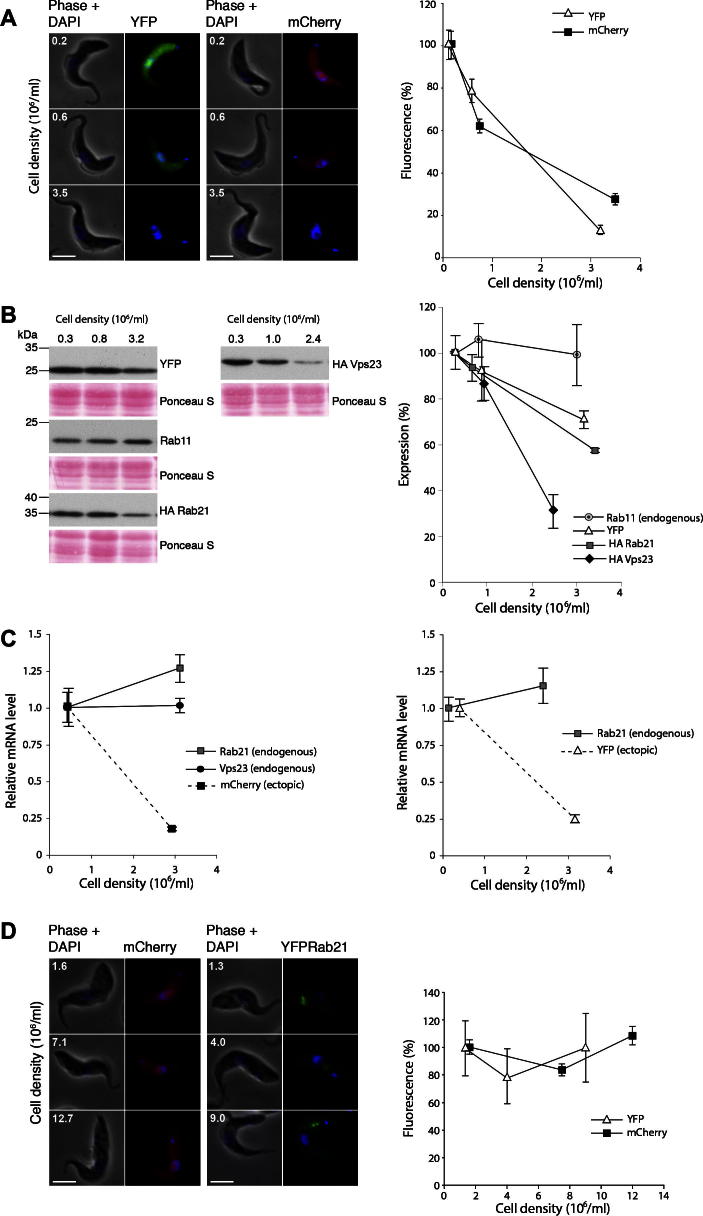
Cell density-dependent ectopic expression in BSF cells. (A) BSF cells expressing YFP and mCherry were fixed at various cell densities and amount of fluorescence quantified by single cell analysis using intrinsic fluorescence. Quantitation (*n* = 30) at right. (B) Western blot analysis of BSF cells expressing YFP, HA-TbVps23 and HA-TbRab21, with Ponceau S staining of the membranes prior to probing to demonstrate equivalence in loading and used for normalisation. TbRab11 shown as endogenous control, quantitation at right from three independent experiments. (C) mRNA levels for mCherry, YFP, Rab21 and Vps23, quantified by qRT PCR. Data are normalised to TERT and are from two biological replicates amplified in triplicate. (D) PCF cells expressing YFP-Rab21 and mCherry at various cell densities showing stable expression levels (*n* = 30 cells). In all panels, where relevant, error bars show standard error of mean and the scale bar represents 5 μm.

Fluorescent proteins, including yellow fluorescent protein (YFP) and mCherry (monomeric mutant of *Discosoma* sp. Red fluorescent protein) were expressed in bloodstream form (BSF) 427 cells using the pHD1034 vector. pHD1034 is driven by a constitutively active ribosomal RNA promoter and capable of stable chromosomal integration into the ribosomal spacer of *T. brucei* (Quijada et al., 2002). Both YFP and mCherry were located throughout the cytoplasm as expected, and were of the expected molecular weight on Western blotting (Fig. 1). The level of YFP fluorescence in fixed cells decreased roughly by an order of magnitude when low-density cultures (0.2 × 10^6^ cells/ml) were grown to reach a higher density of approximately 3 × 10^6^/ml (Fig. 1A). On closer inspection it was apparent that the reduction in fluorescence was not restricted to very high-density cultures but also present in log phase cells. To rule out the possibility that reduced fluorescence in single cell analysis was due to differences in cell volume between low and high density cultures, the perimeter of randomly selected cells were measured. Results showed no apparent differences in cell size in the range of cell densities studied (data not shown). Further, when the amount of ectopically expressed protein was quantified by Western blotting, it was clear that YFP expression levels were also decreased, ruling out differences in fixation or YFP maturation as an explanation for altered fluorescence.

To investigate if the effect were specific to GFP and GFP-variants, N-terminally HA-tagged TbRab21 and mCherry were expressed in BSF 427 cells, again using the pHD1034 vector. Both showed a reproducible reduction in recombinant protein level with increasing density of the cell culture (Fig. 1). These data suggest that decreased expression is not specific to GFP or variants, or to the approach used to detect protein levels.

A fourth protein was investigated. C-terminally HA-tagged TbVps23 (the ortholog of mammalian Tsg101) (Leung et al., 2008), expressed using the rRNA promoter based, ribosomal spacer integrating pXS5 vector (Pal et al., 2002). The construct here has a distinct backbone to pHD1034, but uses the same promoter. HA-TbVps23 showed similar density-dependent effects in the BSF SMB cell line. TbRab11 was used as internal control for natively expressed proteins and remained unperturbed at higher cell density (Fig. 1B). The decrease in expression of various proteins as quantified by Western analysis was less than for fluorescent microscopy, but may be explained by lower dynamic range for blotting compared to fluorescence.

Additional proteins, including C-terminal HA-epitope-tagged Rab28 and YFP tagged Rab21 showed similar density-dependent expression (data not shown). Furthermore the effect was not limited to any particular BSF cell line, as the SMB single marker bloodstream cells expressing T7 RNA polymerase (Wirtz et al., 1999) also showed similar effects when epitope-tagged HA-Rab21 was expressed using pHD1034 (not shown). As the effect is not restricted to overexpressed endogenous proteins, a specific cell line or specific tag it can be concluded that this is a general phenomenon, i.e. reduced rRNA promoter based ectopic expression at higher cell density. Further, analysis of ectopic GFP expression mediated by read through transcription within the tubulin locus did not exhibit the density phenomenon, suggesting that the effect is specific to the rRNA-based vectors (M. Carrington, personal communication).

### 3.2 Density-dependent expression is at the mRNA level and BSF-specific

To investigate whether reduced levels of ectopically expressed proteins in high density BSF cells was due to reduced mRNA levels or protein translation efficiency, mRNA levels of ectopically expressed genes were quantified by qRT-PCR. A well characterised control was chosen for accurate quantification (Brenndorfer and Boshart, 2010). mRNA levels for β-tubulin, PFR2 and telomerase reverse transcriptase (TERT) were compared; expression stability M values were calculated as average pair wise variation for a particular reference gene with all other tested reference genes (Vandesompele et al., 2002; Brenndorfer and Boshart, 2010). mRNA levels of ectopically expressed YFP and mCherry (pHD1034, BSF 427) decreased significantly when low and high culture density aliquots from the same culture were compared using TERT as a constitutive control. Steady state mRNA levels of YFP and mCherry decreased fivefold, whereas mRNAs for the endogenous proteins TbRab21 and TbVps23, i.e. unmanipulated and expressed from their endogenous loci, remained unchanged (Fig. 1C). Therefore density-dependent expression of ectopic proteins can be ascribed at least in part to decreased mRNA copy number, and not simply decreased translatability or increased protein turnover.

To investigate if altered ectopic expression is restricted to bloodstream form cells or is more generalised ectopic rRNA-promoter based expression was analysed in PCF cells. As the pHD1034 vector is suitable for expression in both life stages the same constructs that were used in BSF for mCherry and YFP-tagged Rab21 were used to express these fusion proteins in PCF. Cells were fixed at different densities taken from the same culture and analysed to determine mean native fluorescence. Reproducible results were obtained from independent replicates, and the mean fluorescence of mCherry and YFP remained unchanged across a culture density range of 1 × 10^6^ cells/ml to 10 × 10^6^ cells/ml (Fig. 1D). The level of ectopic YFP-tagged TbRab21 also remained unaltered when analysed by Western blotting. Similarly mRNA levels, as analysed by qRT-PCR, remained unperturbed between low and high density PCF cultures (data not shown). These data suggest that the density-dependent effect is restricted to the BSF.

### 3.3 Density-dependent expression is likely due to trans-acting secreted factors

A possible mechanism for reduced expression at high culture density could be pericellular hypoxia or localised nutrient depletion. As cells settle to the bottom of flasks if not agitated this can lead to transcriptional changes, for example up regulation of the trypanosome transferrin receptor (Mussmann et al., 2004).

BSF cells expressing HA-tagged TbVps23 in the pXS5 vector or YFP/mCherry in pHD1034 were grown in spinning culture flasks (CELLSPIN system, Integra Biosciences, Switzerland) and expression analysed by qRT-PCR, Western blotting and fluorescence microscopy. As a control transferrin receptor levels were analysed by Western blot in cells from different culture densities. The transferrin receptor was expressed at low levels in spinner cultures whereas expression increased several-fold in stationary cultures at high densities (data not shown). This was consistent with previous observations (Mussmann et al., 2004) and also validated our spinner cultures. However expression of the ectopic proteins at higher culture densities was also low in spinner cultures and comparable to the stationary cultures. Therefore pericellular hypoxia or nutrient deficiency due to settling is unlikely the cause of altered expression.

**Fig. 2:**
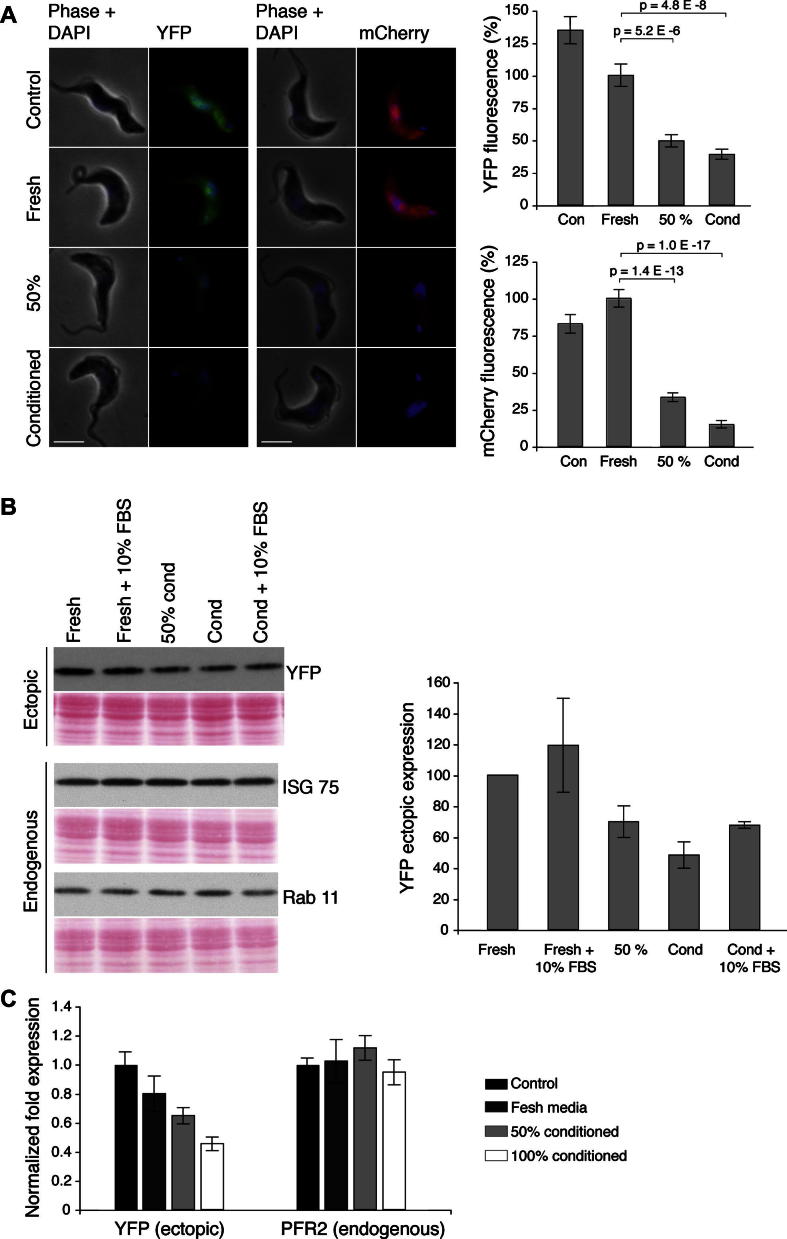
Conditioned media mimic the effect of higher culture density for ectopic expression in BSF cells. (A) YFP and mCherry expressing BSF cells were grown in conditioned media and fluorescence related to recombinant proteins was quantified by microscopy. Right panel shows quantitation from 25 cells. Student’s *t*-test showed significantly reduced expression. (B) Western blot showing reduced level of recombinant YFP in conditioned media grown cells, while the endogenously expressed Rab11 and ISG75 remained unchanged. Right panel shows quantitation based on data from two experiments. (C) The abundance of ectopically transcribed YFP mRNA from BSF cells grown in various media was estimated by qRT PCR. Triplicate results were normalised to beta tubulin and experimental media expression was calibrated against control fresh media, for ectopic YFP and endogenous PFR2. For all panels, error bars shows standard error of mean, where relevant. Scale bar represents 5 μm.

To explore if cells at high density had modified the medium in some manner, cells were grown in the presence of conditioned media obtained from high density monomorphic 427 BSF cultures. BSF cells expressing YFP and mCherry (pHD1034 vector) were grown in 100% conditioned media, 50% conditioned media and fresh media. After 24 h growth in the respective media, cells were fixed and fluorescence from ectopic fluorescent proteins determined. The level of expressed protein from cells grown in conditioned media was significantly lower than cells grown in fresh media, even though the cells in conditioned media grew slowly and were at a lower density compared to cells in fresh media. The level of fluorescence from cells in 50% conditioned media was intermediate between fresh and conditioned media (Fig. 2A). Western blot analysis confirmed these observations and a similar pattern of decreased expression of YFP was observed in conditioned media, while endogenous proteins including ISG75 and Rab11 remained unaffected (Fig. 2B), including ISG75 which has a short half-life of three hours and is therefore likely to be more easily perturbed than a long half-life protein (Leung et al., 2011).

To rule out that reduced expression in conditioned media cells could be due to exhaustion of serum components additional experiments included conditioned media with 10% excess serum and fresh media with 10% excess serum, i.e. 20% total serum. These excess serum cultures followed a similar pattern of reduced expression. YFP mRNA level also decreased in conditioned media as expected, whereas endogenous control mRNAs remained unaltered (Fig. 2C). Overall, these data suggest a possible role for a secreted factor which could be related to stumpy induction factor (SIF) or a similar moiety (Amiguet-Vercher et al., 2004). Monomorphic cells are not believed to be fully responsive to conditioned media or SIF, which suggests that the effect here may not be mediated directly by SIF itself (Vassella et al., 1997); identification of this factor is beyond the scope of the present work. BSF cells at higher cell density showed generally reduced protein translation, as analysed by incorporation of [^35^S] Met/Cys into newly translated proteins; [^35^S] incorporation was reduced to 77% (±s.e. 4.6) and 16% (±s.e. 2.8) as culture density increased from 0.3 × 10^6^/ml to 0.6 and 2.1 × 10^6^/ml. This, however, is insufficient to explain the reductions to ectopic expression, as levels of endogenous short half-life proteins remained essentially unaltered.

## 4 Discussion

We describe here a density-dependent effect on ectopic expression from a variety of proteins driven by rRNA promoter-based vectors. The effect is specific to the bloodstream stage and likely mediated in part by changes to mRNA copy number. Further, we suggest that the effect is probably due a secreted component in media conditioned by parasites grown to high density; supplementation with fresh fetal bovine serum indicates the effect is unlikely a result of depletion of serum components. Similar effects were not found in procyclic forms, nor was a significant effect observed on expression from endogenous loci. Using combined Western analysis, fluorescence and qRT-PCR negates an artefact from the analytical method used for quantitation. Clearly these data have implications for the analysis of ectopically expressed proteins, and especially where an experimenter wishes to determine copy number or to assess the effect of overexpression.

Trypanosomes are the only known eukaryotes where RNA polymerase I transcribes protein coding genes, i.e. VSG and procyclin, in addition to ribosomal genes. While the VSG promoter is distinct from procyclin and rRNA promoters, these all recruit RNA pol I and transcription factors (Brandenburg et al., 2007). This bifunctional RNA polymerase I facilitates very high expression of surface proteins but developmentally regulated in a distinct manner from RNA pol II that transcribes most protein coding genes in an apparently constitutive fashion, albeit with post-transcriptional modulation of mRNA copy number (Clayton, 2002). During development, BSF parasites become heterogeneous, comprising proliferative long slender (LS) forms and cell cycle arrested short stumpy (SS) forms. These SS form cells are pre-adapted for fly transmission and transformation to procyclic forms. It is tempting to speculate that decreased rRNA promoter-based transcription and subsequent translation is a regulatory mechanism used by BSF cells to prepare for differentiation, and that this mechanism is active prior to the cells differentiating into SS form, or that even in monomorphic cells, as studied here, a form of stumpy-like transcriptional behaviour is possible Pays et al., 1993. Furthermore, reduced expression from the rRNA promoter-based reporter constructs following conditioned media exposure is a further indication that monomorphic cells do retain underlying molecular mechanisms to sense and respond to secreted components. As the molecular identification of SIF has yet to be reported, we were unable to directly test SIF and so the identify of the present factor and its similarity with SIF remains unknown. Previous studies were mostly restricted to pleomorphic lines and followed transcriptional changes throughout development from BSF to PCF cells. The categorising of bloodstream strains into pleomorphic or monomorphic itself is not strict as monomorphic lines, though unable to differentiate (Vassella et al., 1997), do retain some of the underlying molecular capability to differentiate (Breidbach et al., 2002). In conclusion, ectopic expression in bloodstream stages is density-dependent, and mechanistically is mediated via mRNA and is a response to in trans factors in the medium of high density cultures. Analysis of copy number of proteins expressed using rRNA based plasmids requires careful attention to cell density and culture conditions for accurate quantitation.
